# Vibronic and Cationic Features of 2-Fluorobenzonitrile and 3-Fluorobenzonitrile Studied by REMPI and MATI Spectroscopy and Franck–Condon Simulations

**DOI:** 10.3390/molecules28124702

**Published:** 2023-06-12

**Authors:** Shuxian Li, Yan Zhao, Yuechun Jiao, Jianming Zhao, Changyong Li, Suotang Jia

**Affiliations:** 1 State Key Laboratory of Quantum Optics and Quantum Optic Devices, Institute of Laser Spectroscopy, Shanxi University, Taiyuan 030006, China; 2 Department of Physics and Electronics Engineering, Jinzhong University, Jinzhong 030619, China; 3 Collaborative Innovation Center of Extreme Optics, Shanxi University, Taiyuan 030006, China

**Keywords:** fluorobenzonitrile, vibronic spectroscopy, cationic spectroscopy, MATI, Franck–Condon simulation

## Abstract

Fluorinated organic compounds have superior physicochemical properties than general organic compounds due to the strong C-F single bond; they are widely used in medicine, biology, pesticides, and materials science. In order to gain a deeper understanding of the physicochemical properties of fluorinated organic compounds, fluorinated aromatic compounds have been investigated by various spectroscopic techniques. 2-fluorobenzonitrile and 3-fluorobenzonitrile are important fine chemical intermediates and their excited state S_1_ and cationic ground state D_0_ vibrational features remain unknown. In this paper, we used two-color resonance two photon ionization (2-color REMPI) and mass analyzed threshold ionization (MATI) spectroscopy to study S_1_ and D_0_ state vibrational features of 2-fluorobenzonitrile and 3-fluorobenzonitrile. The precise excitation energy (band origin) and adiabatic ionization energy were determined to be 36,028 ± 2 cm^−1^ and 78,650 ± 5 cm^−1^ for 2-fluorobenzonitrile and 35,989 ± 2 cm^−1^ and 78,873 ± 5 cm^−1^ for 3-fluorobenzonitrile, respectively. The density functional theory (DFT) at the levels of RB3LYP/aug-cc-pvtz, TD-B3LYP/aug-cc-pvtz, and UB3LYP/aug-cc-pvtz were used to calculate the stable structures and vibrational frequencies for the ground state S_0_, excited state S_1_, and cationic ground state D_0_, respectively. Franck–Condon spectral simulations for transitions of S_1_ ← S_0_ and D_0_ ← S_1_ were performed based on the above DFT calculations. The theoretical and experimental results were in good agreement. The observed vibrational features in S_1_ and D_0_ states were assigned according to the simulated spectra and the comparison with structurally similar molecules. Several experimental findings and molecular features were discussed in detail.

## 1. Introduction

Due to the presence of the strong C-F single bond within the molecule, fluorinated organic compounds have superior physicochemical properties and are widely used in medicine, biology, pesticides, and materials science [1,2,3,4,5]. In recent years, a large number of fluorinated aromatic compounds have been investigated by various spectroscopic techniques. Ling et al. used femtosecond time-resolved photoelectron imaging to study the conformation of bi-fluorophenol and bi-fluoroaniline in the excited state S_1_ after photoexcitation [6,7]. Wijngaarden’s group used high-resolution microwave spectroscopy to measure the rotation spectra of fluorine substituted benzaldehyde, benzonitrile, phenol, and pyridine derivatives to study the molecular structure changes caused by fluorination and intramolecular hydrogen bonding interactions [8,9,10]. Many experimental groups have also studied the vibrational spectra of excited state S_1_ and cationic ground state D_0_ of fluorine-substituted phenol, anisole, and aniline derivatives using laser-induced fluorescence (LIF), resonance-enhanced multiphoton ionization (REMPI), and mass-analyzed threshold ionization (MATI) spectroscopy [11,12,13,14]. Mono-fluorobenzonitrile is a very important class of intermediate for organic synthesis; its vibrational and rotational properties have been reported in many studies [15,16,17,18]. Kamaee et al. investigated the structural trends in mono-, di-, and pentafluorobenzonitriles using Fourier transform microwave spectroscopy [10]. Palmer et al. measured photoelectron spectroscopy of 2-fluorobenzonitrile (2FBN) and 3-fluorobenzonitrile (3FBN) and reported the ionization energies (IEs) of 9.78 eV and 9.79 eV, respectively [19]. Jiang and Levy used laser-induced fluorescence and dispersive fluorescence spectroscopy to study the vibrational relaxation of the excited state of 4-fluorobenzonitrile (4FBN) molecule [20]. In 2018, Zhao et al. [21] studied the vibrational features of the excited and cationic ground states of 4FBN by REMPI and MATI techniques. Silva et al. [16] measured the UV-Vis spectra of monofluorobenzonitriles in dichloromethane; from the curves they measured, the approximate origins of 2FBN and 3FBN could be estimated at 283 nm. To the best of our knowledge, the vibrational properties of the excited states and cationic ground states of 2FBN and 3FBN have not been reported in the literature. 

MATI and zero kinetic energy (ZEKE) spectroscopy are currently the most popular high-resolution techniques for measuring vibrational features of cationic ground states. Kwon’s group built a vacuum ultraviolet single photon MATI system to study many cationic vibrational features [22,23,24,25,26,27,28,29]. Tzeng’s group and Ketkov’s group used two-color MATI to study the cationic spectra of many benzene derivatives and sandwich molecules [30,31,32,33,34,35,36]. Wright’s group used ZEKE technology to research cationic vibrational features of many halogenated benzene and their derivatives [37,38,39,40,41,42]. In this paper, we used two-color REMPI and MATI techniques to study the vibrational features of the excited states and cationic ground states of 2FBN and 3FBN. The precise excitation energies and adiabatic ionization energies were determined. The measured vibrational features were assigned and several experimental findings were analyzed and discussed in detail.

## 2. Results

**Figure 1:**
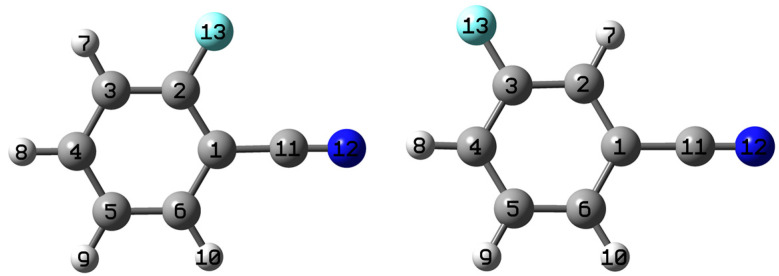
The stable structures of 2- and 3-fluorobenzonitrile with atomic labels.

The stable structures of 2- and 3-fluorobenzonitrile with atomic labels are shown in Figure 1. 2FBN and 3FBN molecules consist of 13 atoms with a total of 33 normal vibrational modes, 30 modes of which are at aromatic ring and 3 modes at CN group. The labeling convention of the vibrational modes followed the Varsanyi system [43]. Vibronic transitions were expressed in the Wilson notation based on the benzene modes, where the v′ ← v″ transition in the normal mode n was represented by $n_{v\prime \prime }^{v\prime }$ [44]; subscript V″ was omitted in the present research as it was a constant 0 (the low energy level of the transition is the vibrationless or zero point energy level of the low electronic state).

### 2.1. Vibronic Features of 2-Fluorobenzonitrile in the S_1_ State

**Figure 2:**
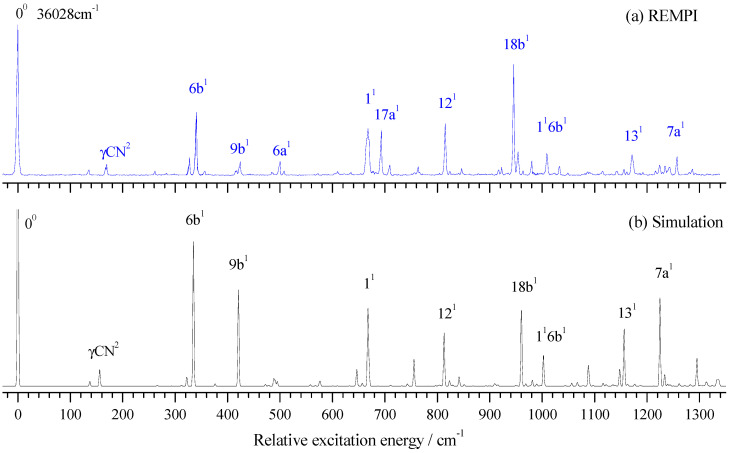
REMPI spectrum of 2-fluorobenzonitrile (**a**) and its Franck–Condon simulation (**b**).

The vibronic spectrum of the S_1_ ← S_0_ transition of 2FBN was measured by a two-color REMPI experiment with the vibration frequency range of 0–1350 cm^−1^. The experimental result is shown in Figure 2a and its Franck–Condon simulation calculated at TD-B3LYP/aug-cc-pvtz level is shown in Figure 2b. It can be seen that the experimental result was in good agreement with the calculated one. The obvious feature of both REMPI and its simulation in Figure 2 was that the rate of signal-to-noise in the low frequency region was greater than in the high frequency region. The simulation spectrum showed that the bands in the high frequency region were dense and consisted of many fundamentals, overtones, and combinations of various modes, many of which were very weak. Thus, dense and weak bands raised the spectral baseline and resulted in a bad rate of signal-to-noise in high frequency regions. The band at certain frequencies in the spectrum maybe came from several component (or vibration mode) contributions. For simplicity’s sake, we only list the largest contributor in Table 1.

Based on DFT calculation and spectral simulation, we analyzed and assigned the vibronic spectra of 2FBN. It is very clear in Figure 2a that the band at 36,028 cm^−1^ was assigned to the band origin of the S_1_ ← S_0_ transition. Many in-plane vibrational modes of the ring were active and most of them were very strong in the REMPI spectrum. The bands at 136, 341, 424, 500, 668, 815, 946, 1171, and 1257 cm^−1^ were assigned to fundamental modes 15, 6b, 9b, 6a, 1, 12, 18b, 13, and 7a, respectively. One out-of-plane fundamental mode at the ring was observed, which appeared at 693 cm^−1^ and was assigned to mode 17a. Several overtone vibrations were observed, which appeared at 170, 635, and 846 cm^−1^ and assigned to γCN^2^, 16b^2^, and 9b^2^, respectively. Other bands observed in the REMPI spectrum were assigned to the combined vibrations of several modes. All the measured vibrational frequencies, calculated frequencies, and possible assignments are listed in Table 1.

From the measured REMPI spectra in Figure 2a, we found that the vibronic band 1^1^ was much wider than other bands. From the simulation calculation, we knew that the band 1^1^ consisted of three components: 16a^1^10a^1^ (665.2 cm^−1^), 1^1^ (667.5 cm^−1^), and 6b^2^ (669.7 cm^−1^). The calculated dipole strengths at the level of TD-B3LYP/aug-cc-pvtz for these three components were 3.365 × 10^−5^, 1.254 × 10^−2^, and 3.2 × 10^−3^, respectively. Due to the very close vibrational energy, the resonance interactions may have played a role in the broadening of the experimental spectral line.

### 2.2. Photoionization Efficiency (PIE) Spectra of 2FBN

**Figure 3:**
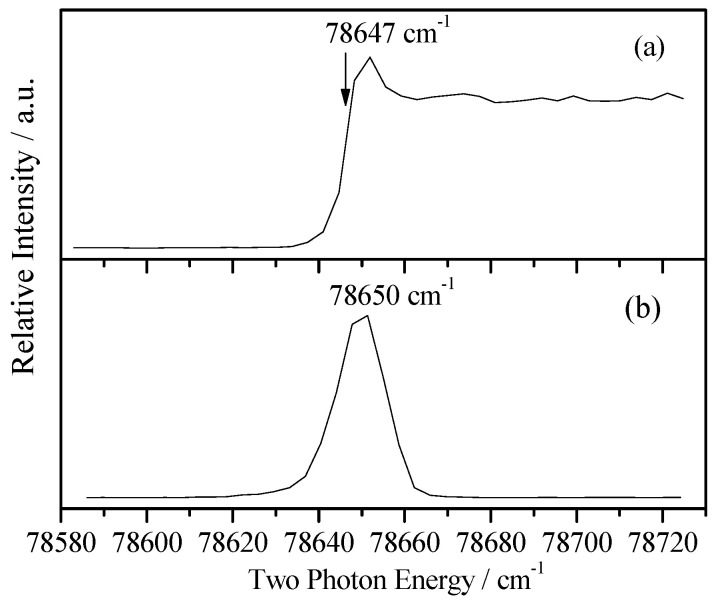
PIE spectrum of 2-fluorobenzonitrile recorded by ionizing via S_1_0^0^ intermediate state at 36,028 cm^−1^ (**a**) and MATI spectrum near the cationic origin 0^+^ for comparison (**b**).

In order to measure the cationic spectra, we first required to know the ionization energy (IE). With the present experimental setup, the IE could be measured by photoionization efficiency (PIE) or MATI experiments. The PIE approach detected the prompt ions involving the field-ionization of high Rydberg neutrals and yielded a strong signal that led to an abruptly rising step near the ionization limit. In contrast, the MATI spectrum detected the threshold ions and yielded a sharp peak at the ionization threshold and vibrational features of the cation. We recorded both the PIE and MATI spectra by scanning the frequency of the ionization laser over a large range to determine the IE of 2FBN. Figure 3a,b show the PIE and MATI spectra via the intermediate state S_1_0^0^ (36,028 cm^−1^). The adiabatic IE of 2FBN was determined to be 78,647 ± 10 cm^−1^ by PIE and 78,650 ± 5 cm^−1^ (9.7514 ± 0.0006 eV) by MATI, including the correction of the Stark effect, respectively. These results were in good agreement with the previous measured value of 9.78 eV (78,881 cm^−1^) [19] by photoelectron spectroscopy with an He I UV-light source. 

### 2.3. Cationic Spectra of 2FBN

To investigate the molecular geometry and vibrational features of the 2FBN cation, the MATI spectra were recorded by ionizing via the S_1_0^0^, S_1_6b^1^ (0^0^ + 341 cm^−1^), S_1_1^1^ (0^0^ + 668 cm^−1^), S_1_12^1^ (0^0^ + 815 cm^−1^), and S_1_18b^1^ (0^0^ + 946 cm^−1^) intermediate states. 

**Figure 4:**
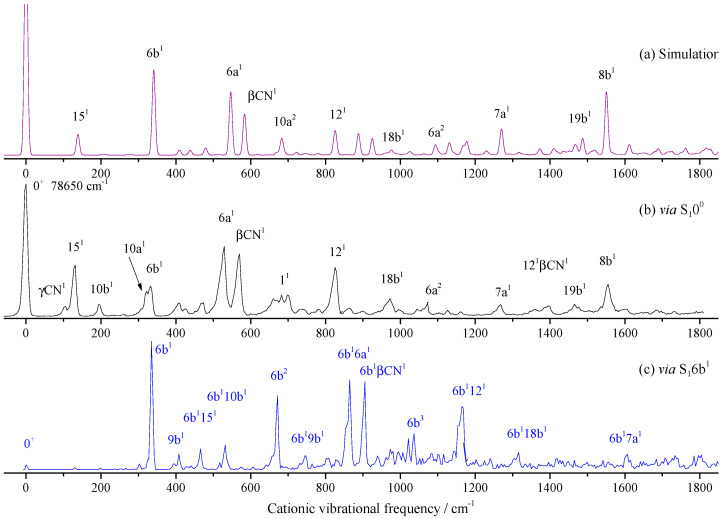
Franck–Condon simulation of the D_0_ ← S_1_0^0^ transition (**a**) and the MATI spectra of 2-fluorobenzonitrile *via* S_1_0^0^ (**b**) and S_1_6b^1^ (**c**) intermediate states.

We first performed the theoretical calculation and spectral simulation. The Franck–Condon simulation is shown in Figure 4a; the corresponding MATI spectrum via S_1_0^0^ is shown in Figure 4b. From Figure 4a,b we know that the theoretical and experimental spectra were in good agreement. The most intense peak corresponded to the origin of the D_0_ ← S_1_ transition of 2FBN. Spectral features were assigned, mainly based on DFT calculations, the Franck–Condon simulation, and comparisons with the available data on substituted benzonitriles. Spectral assignment is a very tedious and error prone matter. Accurate assignments can be obtained by high dimensional or even full dimensional vibrational calculations [45,46]. For the present work, we used the Franck–Condon simulation, which greatly facilitated the spectral identification work. The bands at 131, 333, 530, 571, 683, 826, 972, 1268, and 1555 cm^−1^ were relatively intense and assigned to ring or CN group in-plane motion modes 15, 6b, 6a, βCN, 1, 12, 18b, 7a, and 8b, respectively. Several out-of-plane bending vibrations were also observed, such as γCN and 10b, appearing at 106 and 197 cm^−1^, respectively. Other bands were weak and assigned to overtone or combination vibrations. The measured and calculated cationic vibrational frequencies and their possible assignments are listed in Table 2.

In order to find more vibrational modes of 2FBN cation, the different intermediate states were used to record the MATI spectra. Figure 4c shows the MATI spectra *via* S_1_6b^1^ (0^0^ + 341 cm^−1^). In comparison with Figure 4b, we found that, when S_1_6b^1^ was used as the intermediate state, most of the spectral features could be assigned to combinations of 6b and the modes found in MATI via S_1_0^0^. This could be verified by shifting Figure 4c to the left to align its band 6b with the 0^+^ band in Figure 4b. No more fundamental modes than the MATI via S_1_0^0^ were found.

**Figure 5:**
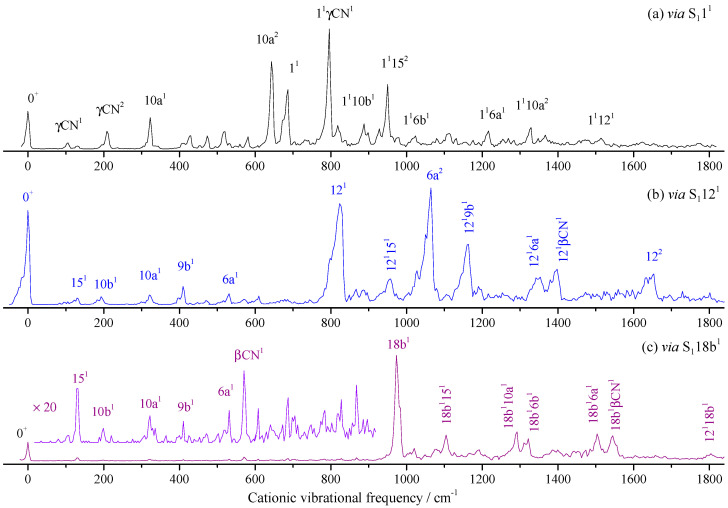
MATI spectra of 2-fluorobenzonitrile *via* S_1_1^1^ (**a**), S_1_12^1^ (**b**), and S_1_18b^1^ (**c**) intermediate states.

Figure 5 shows the MATI spectra via the intermediate states of S_1_1^1^ (0^0^ + 668 cm^−1^), S_1_12^1^ (0^0^ + 815 cm^−1^), and S_1_18b^1^ (0^0^ + 946 cm^−1^). Similarly, when S_1_1^1^ (0^0^ + 668 cm^−1^) was used as the intermediate state, a lot of bands were assigned to the combination vibrations of the mode 1 and those found in MATI via S_1_0^0^. In the lower frequency region, substituent CN out-of-plane bending γCN and its overtone γCN^2^ were found. Aromatic ring out-of-plane bending 10a and its overtone 10a^2^ were also observed. Other bands were weak and were assigned to combination vibrations of several modes.

Similarly, when S_1_12^1^ was used as the intermediate, as shown in Figure 5b, except for the bands at 1064 and 1646 cm^−1^ being assigned to 6a^2^ and 12^2^, other bands greater than 823 cm^−1^ (D_0_12^1^) were assigned to combinations of 12^1^ and other modes. In lower frequency regions, some fundamental modes were active, which were found in the MATI spectrum via S_1_0^0^ or S_1_1^1^. For the MATI via S_1_18b^1^ in Figure 5c, the spectral feature was similar to the MATI via S_1_12^1^; all the assignments, as well as the calculated and measured values, are listed in Table 2.

### 2.4. Vibronic Features of 3-Fluorobenznitrile in the S_1_ State

**Figure 6:**
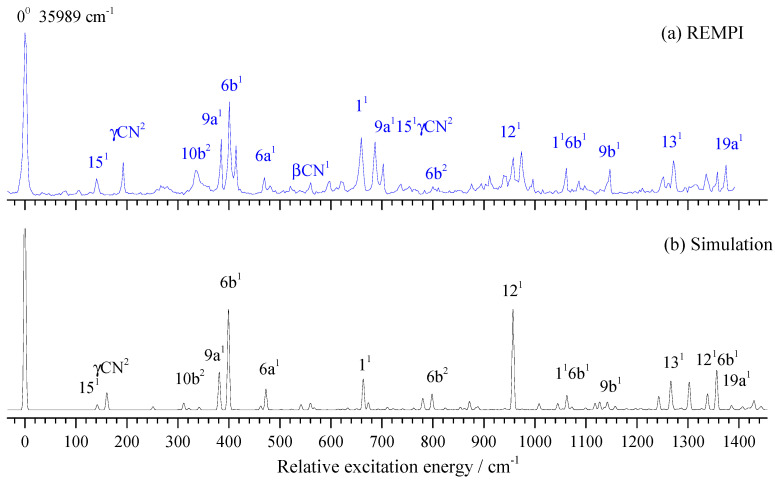
REMPI spectrum of 3-fluorobenzonitrile (**a**) and its Franck–Condon simulation (**b**).

The vibronic spectrum in the S_1_ state of 3FBN is shown in Figure 6a, together with its Franck–Condon simulation shown in Figure 6b for comparison. The entire simulated spectra appeared comparable to the 2-color REMPI spectra in Figure 6a. The distinct band corresponding to the transition energy of 35,989 cm^−1^ was identified as the origin of the S_1_←S_0_ electronic transition. Table 3 lists the observed vibronic transition energies, along with the energy shifts with respect to the band origin, band relative intensities, and possible assignments. The spectral assignment of 3FBN was accomplished by comparing with those of 4-fluorobenzonitrile, 3-fluorophenol, TD-B3LYP/aug-cc-pvtz calculation, and the Franck–Condon simulation. The spectral features in Figure 6a mainly resulted from vibronic transitions related to the in-plane ring deformation and substituent sensitive bending vibrations. The bands appearing at 140, 385, 401, 470, 560, 660, 958, 1147, 1271, and 1374 cm^−1^ were assigned to in-plane stretching or bending vibrations 15, 9a, 6b, 6a, βCN, 1, 12, 9b, 13, and 19a, respectively. The out-of-plane overtone vibrations γ(CN)^2^ and 10b^2^ were also observed in lower frequency regions. Other bands were assigned to combination vibrations of several modes.

### 2.5. PIE Spectra of 3FBN

**Figure 7:**
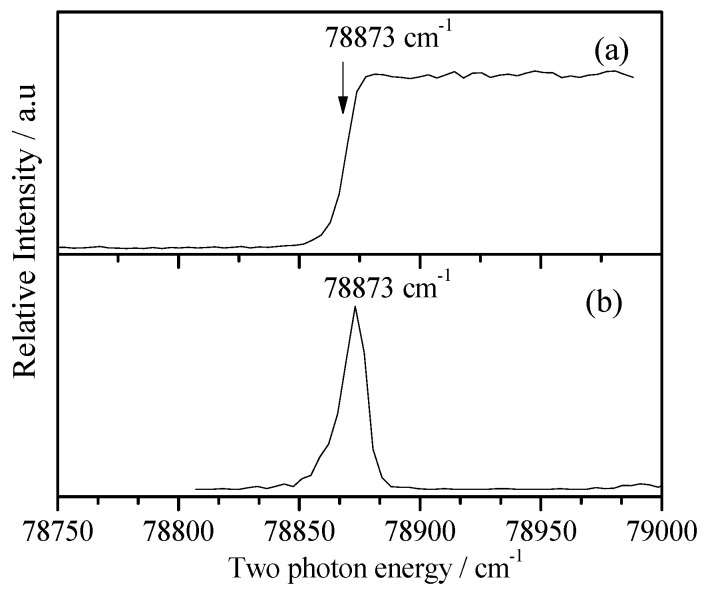
PIE spectrum of 3-fluorobenzonitrile recorded by ionizing via its S_1_0^0^ state at 35,989 cm^−1^ (**a**) and MATI spectrum near the cationic origin 0^+^ for comparison (**b**).

Similar to the 2FBN, ionization energy was very important for the cationic spectral measurements. We first performed the PIE experiment to determine the IE of 3FBN to be 78,873 ± 10 cm^−1^. Then, we measured the MATI spectra to give the precise IE of 3FBN to be 78,873 ± 5 cm^−1^. The PIE and MATI spectra via S_1_0^0^ are shown in Figure 7a,b for comparison. It was obvious that they were very consistent.

### 2.6. MATI Spectra of 3FBN

**Figure 8:**
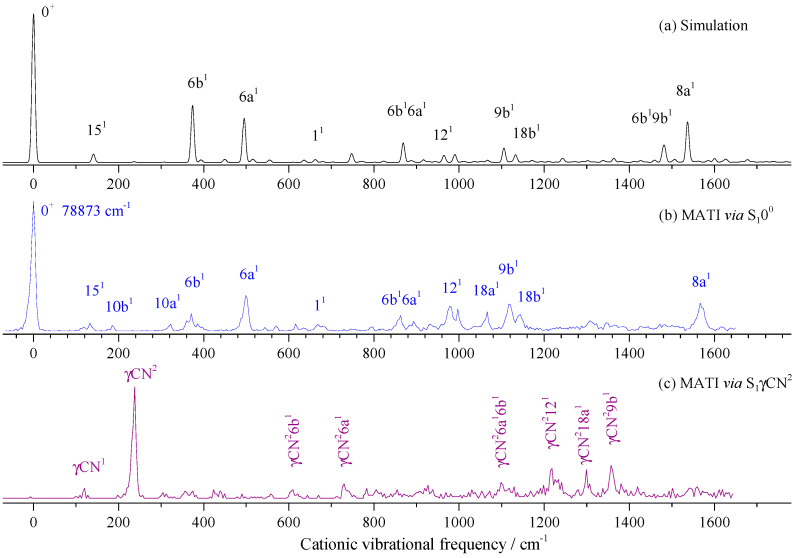
Franck–Condon simulation of the transition D_0_ ← S_1_0^0^ (**a**) and the MATI spectra of 3-fluorobenzonitrile *via* S_1_0^0^ (**b**) and S_1_γCN^2^ (**c**) intermediate states.

Figure 8a,b show the calculated Franck–Condon spectrum and measured MATI spectrum via S_1_0^0^ state at 35,989 cm^−1^, respectively. We can see that they were in good agreement. Many in-plane vibrations were active, such as modes 15, 6b, 6a, 1, 12, 18a, 9b, 18b, 13, and 8a appearing at 133, 371, 498, 668, 978, 1066, 1117, 1144, 1307, and 1566 cm^−1^, respectively. Out-of-plane bending modes 10b and 10a were also observed, but they were weak. Other bands were assigned to combinations of several modes. All the experimental and calculated cationic vibrational frequencies of 3FBN and corresponding assignments are listed in Table 4.

When measuring the MATI via S_1_γCN^2^ (Figure 8c), the distinct feature at 238 cm^−1^ was assigned to D_0_γCN^2^, which followed the propensity rule Δν = 0. The fundamental vibration γCN^1^ was also observed at 120 cm^−1^, with a weak intensity, which did not appear in the REMPI spectrum. Other bands were assigned to combination vibrations of γCN^2^ and fundamental vibrations.

**Figure 9:**
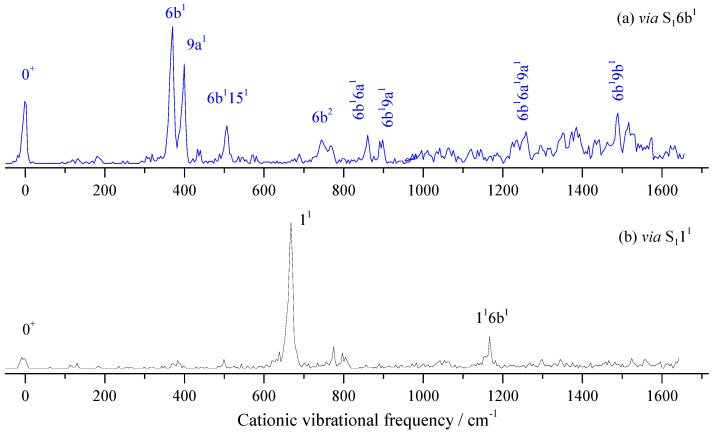
The MATI spectra of 3-fluorobenzonitrile *via* S_1_6b^1^ (**a**) and S_1_1^1^ (**b**) intermediate states.

When measuring the MATI via S_1_6b^1^, as shown in Figure 9a, the distinct feature at 370 cm^−1^ was assigned to D_0_6b^1^, which followed the propensity rule Δν = 0. The intense band at 399 cm^−1^ was assigned to 9a^1^. Other bands were assigned to combination vibrations of 6b^1^ and fundamental vibrations. Figure 9b shows the MATI spectrum via S_1_1^1^, where the cationic vibration 1^1^ (668 cm^−1^) was most intense. The bands at 775, 803, and 1166 cm^−1^ were assigned to combination vibrations 4^1^10b^1^, 1^1^15^1^, and 1^1^6b^1^, respectively.

## 3. Discussion

### 3.1. Breathing Vibrational Band of 2FBN

Whether the vibrational spectra of excited state S_1_ or cationic ground state D_0_, the frequencies of different vibrations in the high-frequency region may have been very close or even the same, which may have come from the fundamental, overtone, or combination vibrations. For example, the breathing vibration 1^1^ of 2FBN in the REMPI spectrum (see Figure 2a) appeared at 668 cm^−1^. The theoretical calculation showed that there were also two weaker vibrations 16a^1^10a^1^ and 6b^2^, whose vibrational frequencies were close to that of mode 1^1^. The calculated vibration frequencies of 16a^1^10a^1^, 1^1^, and 6b^2^ were 665.2 cm^−1^, 667.5 cm^−1^ and 669.7 cm^−1^, respectively. They were so close that the spaces between them were less than the experimental resolution, which led to a wide spectral band in the REMPI spectrum. When using this band as the intermediate state to perform the MATI experiment, according to the propensity rule of Δν = 0, these three vibrational modes of cation may be observed with great intensity. Generally, the vibration frequency of cation is slightly different from that of the excited state for the same vibrational mode and the frequency change can be not consistent for various vibration modes. So, these three vibration modes of cation of 2FBN may be separated in the MATI spectrum. As shown in Figure 5a, the cationic mode 1^1^ appeared at 687 cm^−1^, 6b^2^ appeared at 672 cm^−1^, and 10a^1^ and 10a^2^ were also observed at 322.7 and 643.2 cm^−1^, respectively. The strength of the MATI signal was not only related to the Franck–Condon factor but also to the population of the intermediate state S_1_ and further related to the resonance degree of each vibration mode with the excitation (S_1_ ← S_0_) photon frequency. The experimental results demonstrated that the superposition band of several vibrations could be used as an intermediate state to perform the MATI experiments and more vibrational modes of cation could be observed.

### 3.2. Molecular Structure in S_0_, S_1_, and D_0_ States and Vibrational Frequencies

Theoretical calculations showed that the stable configurations of the ground state S_0_, excited state S_1_, and cationic ground state D_0_ of 2FBN and 3FBN molecules all had Cs symmetry and all the atoms were in the ring plane. This was consistent with their large Franck–Condon factors, intense REMPI and MATI signals, and MATI spectra, following the propensity rule of Δν = 0. However, in the transitions of S_1_ ← S_0_ and D_0_ ← S_1_, the bond length and bond angle of molecules changed slightly. Table 5 and Table 6 show the bond lengths and bond angles of the S_0_, S_1_, and D_0_ states of 2FBN and 3FBN calculated at levels of RB3LYP/ang-cc-pvtz, TD-B3LYP/ang-cc -pvtz, and UB3LYP/ang-cc-pvtz, respectively. It can be seen that the bond lengths between adjacent carbon atoms of the ring of 2FBN were very close to the corresponding bond lengths of 3FBN. After the transition of S_1_ ← S_0_, each C−C bond length increased, resulting in the perimeters of ring of 2FBN and 3FBN increasing by 0.160 Å and 0.158 Å, respectively. The transition of D_0_ ← S_1_ led to the shortening of four C−C bonds and the lengthening of two C−C bonds. The overall effect of D_0_ ← S_1_ transition was that the perimeters of ring of 2FBN and 3FBN decreased by 0.073 Å and 0.072 Å, respectively. Further, the ring C−C bond lengths of D_0_ state was averagely larger than that of S_0_ state. The perimeters of the aromatic ring of 2FBN and 3FBN at the cationic ground states were 0.087 Å and 0.086 Å larger than those of the neutral ground state S_0_, respectively. That is, the perimeters or average bond lengths of the ring in the ground state S_0_, excited state S_1_, and cationic ground state D_0_ met the relationship: S_0_ < D_0_ < S_1_. The length of a chemical bond reflects, to some extent, the strength of that bond. The greater the bond length, the weaker the bond strength. The frequency of an ideal oscillator is proportional to the square root of the bond strength, so the larger the bond length, the lower the vibration frequency. On this basis, we could predict that, on average, the vibration frequencies of the ground state S_0_, the excited state S_1,_ and the cationic ground state D_0_ met the relationship: S_0_ > D_0_ > S_1_. The 33 normal vibration frequencies calculated at the B3LYP/ang-cc-pvtz level of 3FBN were statistically analyzed. On average, the vibration mode frequency of the ground state S_0_ was about 21 cm^−1^ greater than that of the cationic ground state D_0_ and the vibration frequency of D_0_ was about 43 cm^−1^ greater than that of S_1_. For example, the frequencies of breathing vibration mode 1 for S_0_, D_0_, and S_1_ of 2FBN measured in the experiment were 724 [18], 685, and 668 cm^−1^, respectively; for mode 12, they were 835 [18], 823, and 815 cm^−1^, respectively; for mode 18b, they were 1100 [18], 973, and 946 cm^−1^, respectively. The reported experimental and theoretical data of mFBT and mDFB [47] also indicated that most of the vibrational modes of these two molecules followed this rule. Furthermore, from the above vibration data, we know that the frequency variation was larger for the out-of-plane mode (such as 18b of 2FBN) than for the in-plane mode (such as modes 1 and 12 of 2FBN). Our DFT theoretical results showed that this law held for most vibration modes of benzene derivative.

For 2FBN and 3FBN, the bond lengths of C−N in S_0_ state were equal (1.152 Å), also equal in S_1_ state (1.165 Å), and almost equal in D_0_ state (1.158 and 1.155 Å). This means that the C−N bond was very strong and did not change with the substitution position (ortho- or meta-). The C−F bond length yielded a slight change, with different substitution positions.

The aromatic ring included six bond angles of C−C−C. In the electronic transition, the bond angle of the ring also underwent a certain degree of change. Four angles had a variation of approximately 2–3° and the other two had relatively small changes. In the transitions of S_1_ ← S_0_ and D_0_ ← S_1_, variations of the bond angle and bond length of rings led to the ring expansion and contraction, further activating a large number of in-plane vibration modes. Most of the observed vibronic features in the experiments were assigned to in-plane vibrations, only a few of out-of-plane modes were observed. Many benzene derivative molecules have exhibited such characteristics [48,49,50,51,52,53].

### 3.3. Substitution Effect on Ionization Energy

Molecular IE is an important parameter of molecular characteristics. In order to study the effect of fluorine and CN substitutions on ionization energy, we listed the IEs of benzene [54], fluorobenzene [55], benzonitrile [56], 2-fluorobenzonitrile, 3-fluorobenzonitrile, p-fluorobenzonitrile [21], phenol [57], o-fluorophenol [58], m-fluorophenol [59,60], and p-fluorophenol [11] in Table 7 and divided them into four groups for comparison. First, we found that three molecular IEs reduced with respect to their parent molecules, i.e., for the fluorine substitution, the ionization energy of fluorobenzene, 4-fluorophenol, and 4-fluorobenzonitrile was reduced by 330, 490, and 48 cm^−1^ compared with their parent molecules, respectively, where fluorine played a role of electron donor. However, fluorine-substituted ortho and meta benzonitrile slightly increased the IEs by 160 and 383 cm^−1^, respectively; fluorine-substituted ortho and meta (cis and trans) phenols increased the IEs by 1381, 1563, and 1824 cm^−1^, respectively. For these substitutions, fluorine exhibited electron withdrawing properties. It could be seen that the role of fluorine changed with the characteristics of the parent molecule and different substitution positions. Unlike fluorine substitution, CN-substituted benzene and fluorobenzene at ortho, meta, and para positions increased the ionization energy by 3933, 4423, 4646, and 3773 cm^−1^, respectively, playing a role of strong electron withdrawing.

In addition, we can see from Table 7 that the effects of ortho and meta substitution on ionization energy were very close, while the effect of para substitution was relatively weak. Moreover, the IEs of molecules formed by ortho, meta, and para substitutions met the relative relationship: para < ortho < meta. Most benzene derivative molecules followed this rule.

## 4. Materials and Methods

### 4.1. Experimental Methods

The 2-fluorobenzonitrile and 3-fluorobenzonitrile samples were purchased from J&K Chemical and Sigma-Aldrich company, respectively. They were used without further purification. They were a colorless or a light brown liquid with a purity of 99%. The sample was heated to about 130 °C for 2FBN and 60 °C for 3FBN to obtain sufficient vapor pressure. Then, 3 bar krypton for 2FBN and 2.5 bar argon for 3FBN were used as the carrier gases; they carried the sample molecules into the beam source chamber through a pulse valve of 0.5 mm diameter nozzle (0.8 mm for 3FBN). Then, the molecule beam entered the ionization chamber through a skimmer located 20 mm downstream from the nozzle orifice. The vacuum pressures of the beam source and ionization chambers were ~4 × 10^−4^ Pa and ~6 × 10^−6^ Pa, respectively.

The light source consisted of two sets of dye lasers pumped by YAG lasers. One dye laser (CBR-D-24, Sirah) pumped by a frequency-tripled Nd: YAG laser (Qsmart 850, Quantel) was used as the excitation laser. Another dye laser (Precision Scan-D, Sirah) pumped by another frequency-tripled Nd: YAG laser (Qsmart 850, Quantel) was used as the ionization laser for two-color REMPI or probe laser for MATI experiments. The dyes of coumarin 540A and coumarin 460 or coumarin 480 were used for the excitation and ionization lasers, respectively. The dye laser wavelengths were calibrated by a wavemeter (WS7-60 UV-I). The fundamental outputs of the dye lasers were further frequency-doubled by BBO crystals.

Due to the strong electron-withdrawing ability of the CN group, the transition energies of S_1_ ← S_0_ were lower than those of D_0_ ← S_1_ for 2FBN and 3FBN. Such an energy structure indicated that two sets of light sources were required for the measurement of excited state spectra. In the REMPI experiments, we fixed the ionization laser at 232 nm, then scanned the excitation laser from 265 to 279 nm to obtain vibronic spectra of the first electronically excited state S_1_ for 2FBN and 3FBN.

In the MATI experiments, the molecules in neutral ground state S_0_ were resonantly excited to specific vibronic levels in the S_1_ state, further excited to the high Rydberg state by the probe laser, which had a scanning range of 224–240 nm. A −0.5 V/cm pulsed electric field was applied to remove the prompt ions. After a time delay of about 29 μs, the Rydberg molecules were ionized by a 143 V/cm pulsed electric field. Newly formed threshold ions passed through a 48 cm field-free region to be detected by a microchannel plate (MCP) detector. The signal was collected by a multichannel scaler (SRS: SR430) and recorded by a computer. Each mass spectrum was accumulated for 300 laser shots. The time sequence of the whole system was controlled by a pulse delay generator (SRS: DG645). More details on the experimental system have been described in our previous publications [61,62,63].

### 4.2. Theoretical Methods

All calculations were performed using the Gaussian 16 program package [64]. The geometry optimization and vibrational frequencies of S_0_, S_1_, and D_0_ states were calculated at the levels of RB3LYP/aug-cc-pvtz, TD-B3LYP/aug-cc-pvtz, and UB3LYP/aug-cc-pvtz, respectively. Prior to the experiments, we also used the G4 and CBS-QB3 methods to predict IEs in order to select the appropriate dyes. The theoretical predicted adiabatic ionization energies (AIEs) by CBS-QB3 and G4 for 2FBN were 79,111 and 78,972 cm^−1^, respectively, with relative errors of +0.59% and +0.41%. The predicted AIEs by CBS-QB3 and G4 for 3FBN were 79,480 and 79,043 cm^−1^, respectively, with relative errors of +0.77% and +0.22%. The spectral simulations were performed based on the above B3LYP/aug-cc-pvtz calculations, which provided reliable accuracy. The broadening of the REMPI spectral lines was mainly caused by the Doppler effect, while the width of the MATI spectral lines was mainly caused by the ionization field applied to the Rydberg state. The Gaussian line shape, adiabatic Hessian, and time-independent model were used in constructing the spectra [65]. Combined with the theoretical calculations and simulated spectra, the vibrational features of 2FBN and 3FBN measured by the REMPI and MATI experiments were assigned.

## 5. Conclusions

The high-resolution vibrational spectra of the first electronically excited state S_1_ and cationic ground state D_0_ of 2-fluorobenzonitrile and 3-fluorobenzonitrile were measured by two-color resonance-enhanced multiphoton ionization and mass-analyzed threshold ionization spectroscopy. The precise band origins of S_1_ ← S_0_ transition and adiabatic ionization energies were determined to be 36,028 ± 2 cm^−1^ and 78,650 ± 5 cm^−1^ for 2-fluorobenzonitrile and 35,989 ± 2 cm^−1^ and 78,873 ± 5 cm^−1^ for 3-fluorobenzonitrile, respectively. DFT theory at the level of B3LYP/aug-cc-pvtz was used to calculate the molecular structure, vibrational frequency, and to further perform the Franck–Condon simulations. The theoretical results were in good agreement with the experimental measurements. The vibrational features of S_1_ and D_0_ states were analyzed in detail and assigned.

The MATI spectra followed well the propensity rule Δν = 0, indicating that the molecular structures of the cationic ground states were similar to that of the excited states. The molecular structures and vibration frequencies in S_0_, S_1_, and D_0_ states were discussed in detail. The ring C-C bond lengths in S_0_, S_1_, and D_0_ states averagely obeyed the rule of S_1_ > D_0_ > S_0_. The bond length reflected the bond strength; further, the bond length was related to the vibration frequency. On average, or for most vibrational modes, the vibration frequencies of the ground state S_0_, excited state S_1_, and cationic ground state D_0_ met the relative relationship: S_1_ < D_0_ < S_0_. At the transition of S_1_ ← S_0_ and D_0_ ← S_1_, a lot of vibrational modes associated with ring in-plane distortion were active and only a few out-of-plane fundamental vibrations were observed. The substitution effects of F and CN were discussed. Whether the electron donating group or the electron withdrawing group, the ionization energies of molecules formed by ortho, meta, and para substitutions meet the relative relationship: para < ortho < meta. 

## Figures and Tables

**Table 1:** Observed bands in the vibronic spectrum of 2FBN and their possible assignments ^a^.

Transition Energy (cm^−1^)	Relative Intensity	Shift (cm^−1^)	Calc. (cm^−1^)	Assignment ^b^
36,028	100	0	0	0^0^
36,164	4	136	137	15^1^
36,198	7	170	156	γCN^2^
36,290	3	262		10b^1^γCN^1^
36,356	12	328		10a^1^γCN^1^
36,369	42	341	335	6b^1^
36,452	9	424	421	9b^1^
36,528	9	500	495	6a^1^
36,638	3	610		16a^1^10b^1^
36,663	31	635	646	16b^2^
36,696	30	668	668	1^1^
36,721	30	693	698	17a^1^
36,738	7	710	711	6b^1^10b^2^
36,792	6	764	755	9b^1^6b^1^
36,843	34	815	813	12^1^
36,874	2	846	841	9b^2^
36,945	4	917	910	βCN^1^6b^1^
36,950	5	922	916	6a^1^9b^1^
36,974	74	946	960	18b^1^
36,982	15	954	969	12^1^γCN^2^
37,008	10	980	981	6b^1^16b^2^
37,037	14	1009	1003	1^1^6b^1^
37,062	6	1034	1022	16b^2^10b^2^
37,199	14	1171	1156	13^1^
37,285	12	1257	1224	7a^1^
37,315	4	1287	1295	18b^1^6b^1^

^a^ Experimental values are shifts from 36,028 cm^−1^ and the calculated ones (scaled by 0.9649) are obtained from the TD-B3LYP/aug-cc-pvtz calculations. ^b^ β, in-plane bending; γ, out-of-plane bending.

**Table 2:** Assignment of the observed bands (cm^−1^) in the MATI spectra of 2FBN **^a^**.

Intermediate Levels in the S_1_ State					Calc.	Assignment ^b^
0^0^	6b^1^	1^1^	12^1^	18b^1^		
106		106			104	γCN^1^
131			130	132	139	15^1^
197			193	199	205	10b^1^
		209				γCN^2^
		323	321	322	335	10a^1^
333	335				341	6b^1^
407	408		409	410	410	9b^1^
		430				6b^1^γCN^1^
	466					6b^1^15^1^
474		473				15^2^γCN^2^
		520				10a^1^10b^1^
530			531	532	547	6a^1^
	532					6b^1^10b^1^
		582				6b^1^15^1^γCN^1^
		643				10a^2^
571			570	571	584	βCN^1^
	672	672				6b^2^
683		687		687	696	1^1^
698						16a^1^10b^1^
	746					6b^1^9b^1^
		796				1^1^γCN^1^
		818				1^1^15^1^
826			823		826	12^1^
	865					6b^1^6a^1^
		888				1^1^10b^1^
	906					6b^1^βCN^1^
		950				1^1^15^2^
			955			12^1^15^1^
972				973	976	18b^1^
		1023				1^1^6b^1^
	1036					6b^3^
1059			1064			6a^2^
				1104		18b^1^15^1^
	1164		1160			6b^1^12^1^
		1217				1^1^6a^1^
1268					1271	7a^1^
				1292		18b^1^10a^1^
	1316			1321		6b^1^18b^1^
		1329				1^1^10a^2^
			1349			12^1^6a^1^
			1394			12^1^βCN^1^
1396					1410	12^1^βCN^1^
1466					1466	19b^1^
				1503		18b^1^6a^1^
		1513				1^1^12^1^
				1545		18b^1^βCN^1^
1555					1551	8b^1^
	1607					6b^1^7a^1^
			1646			12^2^

^a^ The experimental values are shifts from 78,650 cm^−1^, whereas the calculated ones are obtained from the B3LYP/aug-cc-pVDZ calculations, scaled by 0.9849. ^b^ β, in-plane bending; γ, out-of-plane bending.

**Table 3:** Assignment of observed bands (cm^−1^) in the 2-color REMPI spectrum of 3FBN ^a^.

Transition Energy	Exp.	Relative Intensity	Calc. ^a^	Assignment ^b^
35,989	0	100		0^0^, band origin
36,129	140	11	142	15^1^
36,182	193	21	171	γ(CN)^2^
36,255	266	7	251	10b^1^γ(CN)^1^
36,324	335	16	342	10b^2^
36,374	385	35	381	9a^1^, β(C-F)
36,390	401	58	399	6b^1^, β(CCC)
36,403	414	31	417	10b^1^γ(CN)^3^
36,459	470	11	473	6a^1^, β(CCC)
36,469	480	6	484	10b^2^15^1^
36,509	520	6	523	9a^1^15^1^
36,549	560	9	567	βCN
36,584	595	9	593	10b^3^γ(CN)^1^
36,611	622	9	623	10a^2^γ(CN)^2^
36,649	660	36	663	1^1^, breather
36,675	686	33	684	9a^1^15^1^γ(CN)^2^
36,692	703	20	709	15^1^β(CN)^1^
36,727	738	8	741	6b^1^10b^2^
36,789	800	6	798	6b^2^
36,864	875	8	872	6a^1^6b^1^
36,900	911	13	915	1^1^10b^1^γ(CN)^1^
36,927	938	13	940	6b^2^15^1^
36,947	958	24	957	12^1^
36,963	974	27	975	1^1^10a^1^γ(CN)^1^
36,985	996	11	996	6a^1^9a^1^15^1^
37,051	1062	17	1063	1^1^6b^1^
37,075	1086	9	1087	4^1^6a^1^γ(CN)^1^
37,136	1147	17	1142	9b^1^
37,241	1252	12	1253	6a^1^6b^1^9a^1^
37,260	1271	22	1266	13^1^
37,304	1315	7	1314	11^1^6b^1^16b^1^
37,324	1335	14	1338	12^1^9a^1^
37,347	1358	15	1356	12^1^6b^1^
37,363	1374	19	1385	19a^1^

^a^ The experimental values are shifts from 35,989 cm^−1^, whereas the calculated ones are obtained from the TD-B3LYP/aug-cc-pVDZ calculations, scaled by 0.9722. ^b^ β, in-plane bending; γ, out-of-plane bending.

**Table 4:** Assignment of observed bands (in cm^−1^) in the MATI spectra of 3FBN **^a^**.

Intermediate Levels in the S_1_ State				Calc.	Assignment ^b^
0^0^	γ(CN)^2^	6b^1^	1^1^		
	120			118	γ(CN)^1^
133				141	15^1^
	238			237	γ(CN)^2^
185				188	10b^1^
322				331	10a^1^
371		370		374	6b^1^, β(CCC)
		399		394	9a^1^
498				495	6a^1^, β(CCC)
		506			6b^1^15^1^
	609				γ(CN)^2^6b^1^
615				605	16a^1^
668			668	679	1^1^, breathing
		688			6b^1^10b^1^15^1^
	730				γ(CN)^2^6a^1^
		744			6b^2^
			775		4^1^10b^1^
			803		1^1^15^1^
863		860			6b^1^6a^1^
893		894			6a^1^9a^1^
978				965	12^1^, β(CCC)
997				990	6a^2^
1066				1067	18a^1^, β(CH)
	1098				γ(CN)^2^6a^1^6b^1^
1117				1105	9b^1^, β(CH)
1144				1133	18b^1^, β(CH)
			1166		1^1^6b^1^
	1218				γ(CN)^2^12^1^
		1235			6b^2^6a^1^
		1258			6b^1^6a^1^9a^1^
	1299				γ(CN)^2^18a^1^
1307				1302	13^1^, β(CH)
		1350			6b^1^12^1^
	1357				γ(CN)^2^9b^1^
		1374			6b^1^6a^2^
		1385		1382	19a^1^
		1489			6b^1^9b^1^
1566				1537	8a^1^, ν(CC)
		1516			6b^1^18b^1^
		1574			6b^1^18a^1^15^1^

^a^ The experimental values are shifts from 78,873 cm^−1^, whereas the calculated ones are obtained from the B3LYP/aug-cc-pVDZ calculations, scaled by 0.9704. ^b^ β, in-plane bending; γ, out-of-plane bending.

**Table 5:** Bond length and bond angle of electronic ground state S_0_, first excited state S_1_, and cationic ground state D_0_ of 2-fluorobenzonitrile calculated at RB3LYP/aug-cc-pvtz, TD-B3LYP/aug-cc-pvtz, and UB3LYP/aug-cc-pvtz levels, respectively.

	S_0_	S_1_	D_0_	Δ(S_1_ − S_0_)	Δ(D_0_ − S_1_)	Δ(D_0_ − S_0_)
**Bond length (Å)**						
C1−C2	1.396	1.436	1.455	0.040	0.019	0.059
C2−C3	1.381	1.406	1.393	0.025	−0.013	0.012
C3−C4	1.389	1.409	1.372	0.020	−0.037	−0.017
C4−C5	1.392	1.408	1.434	0.016	0.026	0.042
C5−C6	1.385	1.421	1.386	0.036	−0.035	0.001
C6−C1	1.401	1.424	1.391	0.023	−0.033	−0.010
C1−C11	1.427	1.395	1.407	−0.032	0.012	−0.020
C11−N12	1.152	1.165	1.158	0.013	−0.007	0.006
C2−F13	1.341	1.324	1.296	−0.017	−0.028	−0.045
**Bond angle (°)**						
C1−C2−C3	122.0	124.7	122.5	2.7	−2.2	0.5
C2−C3−C4	118.8	119.0	117.5	0.2	−1.5	−1.3
C3−C4−C5	120.5	118.1	121.1	−2.4	3.0	0.6
C4−C5−C6	120.0	122.7	121.4	2.7	−1.3	1.4
C5−C6−C1	120.4	120.6	119.0	0.2	−1.6	−1.4
C6−C1−C2	118.3	114.9	118.4	−3.4	3.5	0.1

**Table 6:** Bond length and bond angle of the electronic ground state S_0_, first excited state S_1_, and cationic ground state D_0_ of 3-fluorobenzonitrile calculated at RB3LYP/aug-cc-pvtz, TD-B3LYP/aug-cc-pvtz, and UB3LYP/aug-cc-pvtz levels, respectively.

	S_0_	S_1_	D_0_	Δ(S_1_ − S_0_)	Δ(D_0_ − S_1_)	Δ(D_0_ − S_0_)
**Bond length (Å)**						
C1−C2	1.398	1.425	1.380	0.026	−0.045	−0.018
C2−C3	1.380	1.413	1.393	0.033	−0.019	0.013
C3−C4	1.384	1.406	1.433	0.022	0.027	0.049
C4−C5	1.390	1.404	1.374	0.014	−0.029	−0.016
C5−C6	1.387	1.411	1.395	0.024	−0.016	0.008
C6−C1	1.398	1.438	1.449	0.039	0.010	0.051
C1−C11	1.430	1.397	1.415	−0.033	0.017	−0.015
C11−N12	1.152	1.165	1.155	0.013	−0.009	0.003
C2−F13	1.346	1.330	1.300	−0.016	−0.030	−0.046
**Bond angle (°)**						
C1−C2−C3	118.2	118.4	116.9	0.2	−1.4	−1.3
C2−C3−C4	122.5	125.2	123.4	2.7	−1.7	0.9
C3−C4−C5	118.5	115.9	118.8	−2.6	2.9	0.3
C4−C5−C6	120.6	121.1	119.2	0.4	−1.9	−1.4
C5−C6−C1	119.6	122.2	120.9	2.6	−1.3	1.3
C6−C1−C2	120.4	116.9	120.5	−3.4	3.5	0.1

**Table 7:** Ionization energy of benzene and phenol and their F- and CN-substituted molecules (cm^−1^).

Molecule	IE	ΔIE	Molecule	IE	ΔIE
Benzene ^a^	74,557	0	Benzonitrile ^c^	78,490	0
Fluorobenzene ^b^	74,227	−330	2-Fluorobenzonitrile ^d^	78,650	160
Benzonitrile ^c^	78,490	3933	3-Fluorobenzonitrile ^d^	78,873	383
			4-Fluorobenzonitrile ^e^	78,000	−490
Phenol ^f^	68,625	0			
2-Fluorophenol ^g^	70,006	1381	Fluorobenzene ^b^	74,227	0
3-Fluorophenol, cis ^h,i^	70,188	1563	2-Fluorobenzonitrile ^d^	78,650	4423
3-Fluorophenol, trans ^h,i^	70,449	1824	3-Fluorobenzonitrile ^d^	78,873	4646
4-Fluorophenol ^j^	68,577	−48	4-Fluorobenzonitrile ^e^	78,000	3773

^a^ Ref. [54]. ^b^ Ref. [55]. ^c^ Ref. [56]. ^d^ This work. ^e^ Ref. [21]. ^f^ Ref. [57]. ^g^ Ref. [58]. ^h^ Refs. [59,60]. ^i^ Ref. [60]. ^j^ Ref. [11].

## Data Availability

The data that support the findings of this study are available from the corresponding author upon reasonable request.

## Sample Availability

Samples of 2-fluorobenzonitrile and 3-fluorobenzonitrile are available from commercial sources.
